# Elevated α-defensin levels in plasma and bronchoalveolar lavage fluid from patients with myositis-associated interstitial lung disease

**DOI:** 10.1186/s12890-018-0609-5

**Published:** 2018-03-12

**Authors:** Noriho Sakamoto, Hiroshi Ishimoto, Tomoyuki Kakugawa, Minoru Satoh, Tomoko Hasegawa, Shin Tanaka, Atsuko Hara, Shota Nakashima, Hirokazu Yura, Takuto Miyamura, Hanako Koyama, Towako Morita, Seiko Nakamichi, Yasushi Obase, Yuji Ishimatsu, Hiroshi Mukae

**Affiliations:** 1 Department of Respiratory Medicine, Unit of Translational Medicine, Nagasaki University Graduate School of Biomedical Sciences, 1-7-1 Sakamoto, Nagasaki, 852-8501 Japan; 2 Department of Clinical Nursing, School of Health Sciences, University of Occupational and Environmental Health, 1-1 Iseigaoka, Yahatanishi-ku, Kitakyushu, Fukuoka, 807-0804 Japan; 3 Department of Human, Information and Life Sciences, School of Health Sciences, University of Occupational and Environmental Health, 1-1 Iseigaoka, Yahatanishi-ku, Kitakyushu, Fukuoka, 807-0804 Japan; 4 Department of General Medicine, Unit of Basic Medical Sciences, Nagasaki University Graduate School of Biomedical Sciences, 1-7-1 Sakamoto, Nagasaki, 852-8501 Japan; 5 Department of Cardiopulmonary Rehabilitation Science, Unit of Rehabilitation Sciences, Nagasaki University Graduate School of Biomedical Sciences, 1-7-1 Sakamoto, Nagasaki, 852-8520 Japan

**Keywords:** Bronchoalveolar lavage fluid, Human neutrophil peptide, Idiopathic inflammatory myopathies, Interstitial lung disease

## Abstract

**Background:**

Interstitial lung disease (ILD) is a prognostic indicator of poor outcome in myositis. Although the pathogenesis of myositis-associated ILD is not well understood, neutrophils are thought to play a pivotal role. Neutrophils store azurophil granules that contain defensins, which are antimicrobial peptides that regulate the inflammatory response. Here, we evaluated levels of the human neutrophil peptides (HNPs) α-defensin 1 through 3 in patients with myositis-associated ILD to determine whether HNPs represent disease markers and play a role in the pathogenesis of myositis-associated ILD.

**Methods:**

HNP levels were measured in the plasma and bronchoalveolar lavage fluid (BALF) of 56 patients with myositis-associated ILD and 24 healthy controls by enzyme-linked immunosorbent assay.

**Results:**

Analysis revealed significantly higher HNP levels in plasma and BALF samples from patients with myositis-associated ILD as compared to those of healthy controls; however, plasma HNPs were significantly correlated with total cell counts in BALF. Additionally, BALF HNP levels were positively correlated with serum surfactant protein-A and the percentage of neutrophils in BALF, and BALF HNP levels correlated with the percentage of reticular opacities in high-resolution computed tomography results for patients with anti-aminoacyl-tRNA synthetase (ARS) antibody positive myositis-associated ILD. Survival did not differ between patients with higher and lower levels of plasma and BALF HNPs.

**Conclusions:**

Plasma and BALF HNPs might reflect the disease activities of myositis-associated ILD, especially in patients with anti-ARS antibody positive myositis-associated ILD. However further studies are necessary to clarify whether HNPs represent disease markers and play roles in disease pathogenesis.

## Background

Idiopathic inflammatory myopathies (IIMs) are a heterogeneous group of disorders clinically characterized by chronic muscle weakness, low muscle endurance, and the presence of inflammatory cell infiltrates in muscle tissue [1]. Polymyositis (PM), dermatomyositis (DM), and clinically amyopathic dermatomyositis (CADM) are subsets of IIMs that frequently affect the lungs. Interstitial lung disease (ILD) is a common pulmonary manifestation considered a common cause of morbidity and mortality in myositis [2, 3]. Risk factors for ILD in patients with myositis include genetic predisposition and myositis-specific autoantibodies [4, 5]; however, little is known about the clinical course and pathogenesis of myositis-associated ILD. Cellular profiles in bronchoalveolar lavage fluid (BALF) can be used to help diagnose ILD, but only serve to rule out infection in the current clinical differential diagnosis in patients with myositis [6]. However, some reports suggest that the presence of neutrophils in BALF correlates with poor clinical course [2, 7, 8].

Defensins are small, arginine-rich, cationic peptides that exhibit antimicrobial activity [9]. Human cells express α- and β-defensins, and among the six known α-defensins, human neutrophil peptides (HNPs) 1 to 4 are mainly found in neutrophils, whereas human defensins 5 and 6 are primarily expressed in intestinal Paneth cells and the respiratory and female reproductive tracts [10]. In addition to their antimicrobial functions, defensins might also regulate inflammatory responses [11]. We previously identified elevated plasma and BALF HNP levels in patients with various inflammatory lung diseases, including systemic sclerosis-associated ILD, with these levels correlated with neutrophils in BALF [12–19]. These results indicated that plasma and BALF HNP levels play a pivotal role and might serve as biomarkers of other connective-tissue-disease-associated ILD. Here, we evaluated HNP concentrations in BALF and plasma samples from patients with myositis-associated ILD to determine whether HNPs could be used as markers of myositis-associated ILD.

## Methods

### Study population

The study population comprised 56 patients with myositis-associated ILD and who visited the Department of Respiratory Medicine at Nagasaki University Hospital between 2000 and 2015, as well as 24 healthy volunteers. PM/DM and CADM diagnoses were based on the criteria reported by Bohan and Peter [20] and Sontheimer et al. [21], respectively. ILD was diagnosed by high-resolution computed tomography (HRCT) of the lung, and three of these patients were pathologically diagnosed with fibrotic nonspecific interstitial pneumonia by surgical lung biopsy. BALF and blood samples were collected from each patient during the primary visit and stored at − 20 °C until use. Patients were not under treatment with systemic steroid and/or immunosuppressants at the time of sample collection. All data, including those from pulmonary function tests, arterial blood gas analyses, markers of interstitial pneumonia, such as Krebs von den Lungen 6 (KL-6), surfactant protein (SP)-A, and SP-D expression, as well as survival rates, were obtained from medical records. All healthy controls were asymptomatic, not taking any medication, and had normal chest radiographs. The study protocol was approved by the Human Ethics Review Committee at Nagasaki University School of Medicine, and all participants provided written, informed consent before enrollment.

### Evaluation of ILD

All HRCTs and BALF were obtained at the time of diagnosis and at ~ 4-week intervals. HRCT results for all patients were retrospectively and independently assessed by two pulmonologists (N.S. and H.I.). The extent of visual ground glass opacity, consolidation, reticular opacities, and honeycombing were determined by visually estimating the relative area of abnormality in the upper, middle, and lower zones of each lung to the nearest 10%, as previously described [22, 23]. The upper zone was defined as the area above the level of the carina, the lower zone as the area below the level of the inferior pulmonary vein, and the middle zone as the area between the upper and lower zones. The overall involvement percentage was obtained by averaging the six lung zones, with final involvement obtained by averaging the scores of the two observers. BALF was collected with three instillations of sterile physiological saline (50 mL) through a flexible bronchoscope, as previously described [24]. The collected lavage fluid was passed through two sheets of gauze, centrifuged at 400 *g* for 10 min at 4 °C, and the supernatant stored at − 20 °C until analysis.

### HNP quantification

HNP concentrations in plasma and BALF samples were measured using sandwich enzyme-linked immunosorbent assay (ELISA) kits according to manufacturer protocol (HNP1–3; HyCult Biotechnology, Uden, Netherlands). Plasma samples were diluted 1000-fold prior to analysis. The lower limit of detection was 156 pg/mL.

### Immunoprecipitation (IP)

Sera were analyzed by IP of K562 cell extracts radiolabeled with ^35^S-methionine, and the specificities of autoantibodies were determined using specific reference sera [25].

### Anti-Jo-1 and MDA5 ELISAs

Anti-Jo-1 and –melanoma-differentiation-associated protein 5 (MDA5) antibodies were also tested by ELISA, as previously described [26], using recombinant Jo-1 and MDA5 proteins (0.5 μg/mL; Diarect, Freiburg, Germany) and 1:250 diluted sera. The optical density was measured and converted into units using a standard curve created with a prototype-positive serum.

### Statistical analysis

All values are expressed as the median and inter-quartile range (IQR). Differences between groups were compared using Mann-Whitney *U* tests. Differences among groups were determined using the Kruskal–Wallis test for continuous variables. If a significant difference was found by the Kruskal–Wallis test, multiple comparisons were performed using the Dunn test. Statistical significance was defined as *p* < 0.05. Correlations between parameters were determined by Spearman’s rank correlation coefficient. To account for multiple comparisons, we conducted false-discovery rate (FDR) calculations using the Benjamini–Hochberg procedure [27], with FDR q values of 0.1 considered significant.

## Results

### Patient characteristics

Patient demographics are shown in Table 1 (*n* = 56). Fifteen of the patients were men, and the median age was 60 years. Half of the patients were diagnosed with DM. The myositis-specific autoantibodies anti-ARS (Jo-1, PL-7, PL-12, EJ, OJ, and KS) and anti-MDA5 were detected in 46% and 18% of patients, respectively, as determined by IP and ELISA analysis. Patient laboratory findings are shown in Table 2.

**Table 1:** Characteristics of patients with myositis-associated ILD

Characteristic	N (%)
Gender (male/female)	15/41
Age (y)	60 (range, 50–66)
Clinical diagnosis	
Polymyositis	7 (13%)
Dermatomyositis	28 (50%)
Clinically amyopathic dermatomyositis	21 (38%)
Myositis-specific autoantibodies	
anti-ARS Ab	26 (46%)
anti-Jo-1 Ab	11 (20%)
anti-PL-7 Ab	6 (11%)
anti-KS Ab	3 (5%)
anti-PL-12 Ab	2 (4%)
anti-EJ Ab	2 (4%)
anti-OJ Ab	2 (4%)
anti-MDA5 Ab	10 (18%)
anti-Ku Ab	2 (4%)
anti-TIF1 gamma Ab	2 (4%)
Unknown	16 (29%)
Total	56

**Table 2:** Laboratory findings in patients with myositis-associated ILD

Variables	N	Median	IQR
Laboratory data			
CK (IU/L)	55	150	(66–393)
Aldolase (IU/L)	53	7.4	(5.1–19.0)
AST (IU/L)	55	34	(23–59)
ALT (IU/L)	55	28	(20–46)
LDH (IU/L)	55	302	(239–391)
PaO2 (torr)	44	79.4	(69.6–90.2)
KL-6 (U/mL)	53	1030	(440–1793)
SP-D (ng/mL)	49	204	(95–204)
SP-A (ng/mL)	37	72.7	(55.4–105.1)
Pulmonary function test			
%VC (%)	40	83.6	(67.3–94.7)
FEV1/FEV (%)	40	83.7	(78.8–92.8)
%DLco (%)	40	54.8	(39.0–72.0)
BALF cell findings			
TCC (× 10^5^/mL)	45	3.5	(2.8–5.8)
Macrophages (%)	45	45.7	(34.4–63.9)
Lymphocytes (%)	45	33.4	(17.4–47.5)
Neutrophils (%)	45	7.8	(2.2–15.4)
Eosinophils (%)	45	2.2	(0.9–7.2)
CD4/CD8	43	0.4	(0.2–1.1)

*ALT*: alanine aminotransferase; *AST*: aspartate aminotransferase; *CK*: creatine kinase; *DLco*: diffusing capacity of the lungs for carbon monoxide; *FEV*: forced expiratory volume; *TCC*: total cell count; *VC*: vital capacity

### HNP levels in plasma and BALF samples

**Fig. 1:**
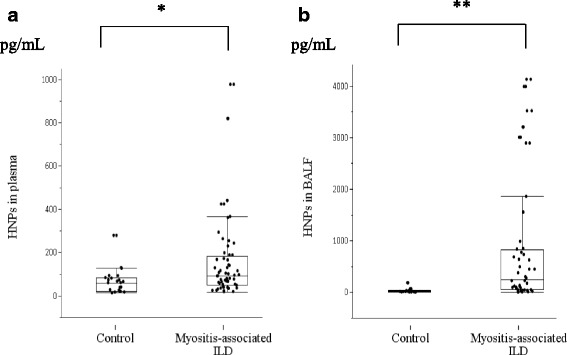
ELISA analysis of patient samples. HNP levels in plasma (**a**) and BALF (**b**) samples from patients with myositis-associated ILD and healthy controls according to ELISA. Boxes represent the IQR, and the internal line represents the median. Whiskers indicate the lowest and highest values within 1.5 × IQR. *p < 0.05; **p < 0.01

**Fig. 2:**
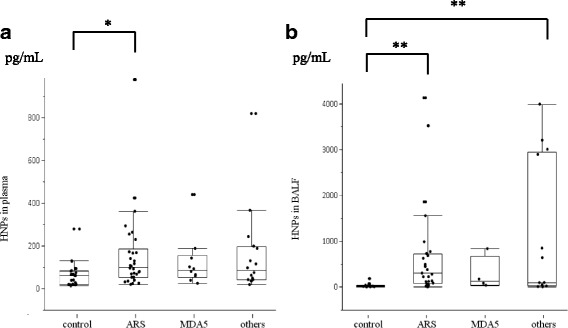
Associations between plasma and BALF HNP levels and patient autoantibody levels. ELISA analysis of associations between HNP levels in the plasma (**a**) and BALF (**b**) and autoantibody levels in patients with myositis-associated ILD and healthy controls. “Others” indicates patients negative for both ARS and MDA5 autoantibodies. *p < 0.05; **p < 0.01

HNP analysis revealed significantly higher levels in the plasma of patients with myositis-associated ILD [78.5 pg/mL (40.1–171.1 pg/mL)] than in that of healthy controls [60.5 pg/mL (20.5–83.3 pg/mL), *p* < 0.05; Fig. 1a], as well as that in the BALF [250.9 pg/mL (53.7–821.0 pg/mL) vs. 14.3 pg/mL (7.8–30.9 pg/mL), *p* < 0.01; Fig. 1b]. Plasma HNP levels were elevated only in patients with ARS autoantibody production; however, significant elevations were observed in BALF samples from both anti-ARS-positive and ARS/MDA5-double-negative subjects (“others”; Fig. 2).

### Association between HNPs and clinical parameters in myositis-associated ILD

**Fig. 3:**
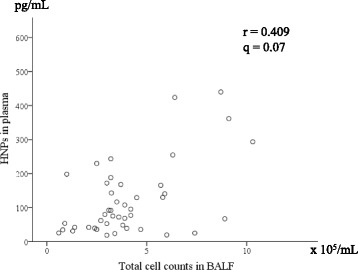
Correlation between plasma HNPs and total cell counts in BALF samples from patients with myositis-associated ILD

**Fig. 4:**
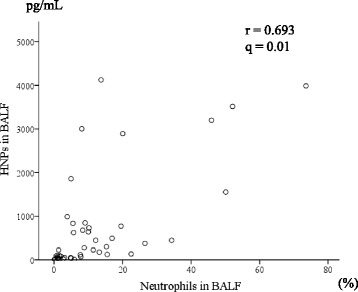
Correlation between HNP levels and neutrophil percentage in BALF samples from patients with myositis-associated ILD

We then analyzed the relationships between clinical parameters and HNP levels in patient plasma and BALF samples (Table 3). Interestingly, the plasma and BALF HNP levels showed no significant association. Plasma HNP levels were significantly correlated with total cell count in BALF (Fig. 3 and Table 2). Moreover, BALF HNP levels were positively correlated with serum SP-A and neutrophil percentage in BALF (Fig. 4 and Table 2). There were no significant associations between HNP levels and pulmonary function test results or HRCT findings. Furthermore, survival determined by Kaplan–Meier survival analysis showed no association with HNP concentration in the plasma or BALF (data not shown).

**Table 3:** Correlation between HNPs and clinical parameters in patients with myositis-associated ILD

	HNPs in plasma					HNPs in BALF		
	N	r	95% CI	*q*-value	N	r	95% CI	*q*-value
Laboratory data								
CK (IU/L)	55	0.135	−0.135–0.387	1.00	43	0.029	−0.274–0.327	0.92
PaO_2_ (torr)	28	−0.062	−0.425–0.318	1.00	35	0.182	− 0.161–0.486	0.49
KL-6 (U/mL)	53	− 0.122	− 0.380–0.153	1.00	41	0.267	−0.044–0.531	0.23
SP-D (ng/mL)	49	0.029	− 0.254–0.308	1.00	38	0.205	−0.123–0.492	0.41
SP-A (ng/mL)	44	0.010	−0.288–0.306	0.95	29	0.528	0.200–0.750	0.02
Pulmonary function test								
%VC (%)	40	−0.023	−0.332–0.291	1.00	34	−0.048	−0.380–0.295	0.93
%DLco (%)	40	−0.071	−0.374–0.246	1.00	34	−0.017	−0.353–0.323	0.92
BALF cell findings								
TCC (×10^5^/mL)	45	0.409	0.131–0.627	0.07	44	0.147	−0.157–0.425	0.49
Lymphocytes (%)	45	−0.013	−0.305–0.282	1.00	44	−0.305	−0.552–0.009	0.17
Neutrophils (%)	45	0.266	−0.030–0.519	0.52	44	0.693	0.499–0.821	0.01
HRCT findings								
Ground glass opacity (%)	52	0.132	−0.146–0.391	1.00	41	0.102	−0.212–0.397	0.69
Consolidation (%)	52	0.095	−0.183–0.359	1.00	41	−0.237	−0.508–0.076	0.30
Reticular opacities (%)	52	−0.028	−0.299–0.247	1.00	41	0.284	−0.026–0.544	0.23

*CI* confidence interval, *CK* creatine kinase, *DLco* diffusing capacity of the lungs for carbon monoxide, *TCC* total cell count, *VC* vital capacity

Next, we examined the association between HNP levels and clinical parameters in each group according to myositis-related autoantibodies (anti-ARS antibody, anti-MDA5 antibody, others). No other significant correlation was evident in each group according to myositis-related autoantibodies other than plasma HNP levels and total cell count in BALF in patients with anti-ARS-antibody positive myositis-associated ILD. On the other hand, BALF HNP levels in patients with anti-ARS-antibody positive myositis-associated ILD were significantly correlated with the extent of reticular opacities and negatively correlated with consolidation according to HRCT findings (Table 4). No other significant correlation was evident in each group categorized by myositis-related autoantibodies other than BALF HNP levels and neutrophil percentage in BALF from patients with anti-ARS-antibody positive myositis-associated ILD. Additionally, there was no significant correlation between HNP levels and clinical parameters in anti-MDA5-antibody positive myositis-associated ILD and ARS/MDA5-antibody double-negative subjects (data not shown).

**Table 4:** Correlation between HNPs and HRCT findings in patients with anti-ARS antibody positive myositis-associated ILD

	HNPs in plasma					HNPs in BALF		
	N	r	95% CI	*q*-value	N	r	95% CI	*q*-value
HRCT findings								
Ground glass opacity (%)	30	0.328	−0.037–0.616	0.49	27	0.085	−0.305–0.451	0.80
Consolidation (%)	30	0.031	−0.333–0.387	1.00	27	−0.452	−0.710−− 0.087	0.08
Reticular opacities (%)	30	−0.089	−0.435–0.280	1.00	27	0.465	0.103–0.718	0.09

*CI* confidence interval

## Discussion

In this study, we found elevated HNP concentrations in the plasma and BALF from patients with myositis-associated ILD as compared with that observed in those from healthy controls. Notably, plasma HNP levels were associated with total cell counts in the BALF, whereas those in BALF samples were associated with the interstitial pneumonia marker SP-A and the percentage of neutrophils in BALF. Furthermore, BALF HNP levels in patients with anti-ARS-antibody positive myositis-associated ILD were significantly correlated with the extent of reticular opacities and negatively correlated with consolidation in HRCT findings.

Previous studies demonstrated that BALF HNP levels are associated with the prevalence of neutrophils in BALF samples and disease activity in patients with various lung diseases, including connective-tissue-disease-associated ILD [14–19]. Moreover, several reports suggest that neutrophils in the BALF correlate with poor clinical course in patients with PM/DM [2, 7, 8]. Consistent with these findings, the present study showed that BALF HNP levels correlated with the amount of neutrophils in the BALF of patients with myositis-associated ILD. Neutrophils release granular and nuclear contents called neutrophil extracellular traps (NETs), including HNPs, in response to different classes of microorganisms, soluble factors, and host molecules [28]. Zang et al. [29] demonstrated that patients with PM/DM have the capacity to form NETs that could not be completely degraded, particularly in patients with PM/DM-ILD. Moreover, they reported that abnormal NET regulation might be involved in PM/DM pathogenesis and could be a factor that initiates and/or aggravates ILD [29]. The authors also reported a higher percentage of low-density granulocytes (LDGs) along with enhanced NET-formation capabilities in patients with DM as compared with healthy controls, and that this percentage was also higher in DM patients with ILD than in those without. Additionally, LDG percentage was positively correlated with lung disease activity scores [30]. In line with these reports, the present results showed that increased HNP levels in the plasma and BALF from patients with myositis-associated ILD suggested that neutrophils are likely to release NETs, including HNPs, which are difficult to degrade in patients with myositis-associated ILD.

Our results also showed that BALF HNP levels correlated with SP-A levels, suggesting the existence of interstitial lung injury [31]; however, this was not observed in HRCT findings for all patients. Additionally, these levels in patients with anti-ARS-antibody positive myositis-associated ILD were significantly correlated with the extent of reticular opacities according to HRCT findings, which we previously reported in patients with systemic sclerosis-associated ILD [19]. Reticular opacities are common HRCT findings in anti-ARS-antibody positive associated ILD with or without myositis [32–34] and reflect fibrosis in anti-ARS-antibody positive ILD [33]. These findings indicate that HNP levels in BALF reflect the fibrotic change in anti-ARS-antibody positive associated ILD. Furthermore, we previously reported that HNPs induce the production of cytokines and growth factors, which act on lung fibroblasts and epithelial cells to induce pulmonary fibrosis and collagen production in vitro [9, 35, 36]. This might suggest that HNPs in the lung both indicate and induce fibrotic change. Further studies are needed to determine whether neutrophil-derived HNPs play a role in the pathogenesis of myositis-associated ILD.

We also observed that plasma HNPs were associated with total cell counts in BALF, which might reflect the lung inflammation observed in patients with myositis-associated ILD. Although the precise mechanism of plasma HNP production is not well understood, these factors are likely derived from neutrophil-precursor cells in the bone marrow following stimulation by inflammatory mediators [18, 37]. Therefore, these results might suggest that lung inflammation elicits increased plasma HNP levels. Nevertheless, further studies are needed to clarify the functional significance of plasma HNPs in patients with myositis-associated ILD.

Although we found that HNP levels were associated with several clinical parameters and suggested to play a role in myositis-associated ILD, it remains unclear whether HNPs can be used as disease markers in myositis-associated ILD. This might be because our definition of myositis-associated ILD included different disease types (PM, DM, or CADM) or the use of myositis-specific antibodies (anti-ARS and anti-MDA5).

The present study has several limitations. First, because the patient population was strictly seen by physicians in the respiratory department, our study only examined HNP levels in patients with myositis-associated ILD, but not those without the ILD component. Therefore, we were unable to confirm that lung pathology was directly responsible for the observed differences in HNP levels. Second, we did not show values for plasma or BALF HNPs, which can discriminate between various types of ILD or infectious processes. We previously reported elevated HNP levels in several types of ILD, as well as according to infectious status [14–19], indicating that increased levels of HNPs were nonspecific in myositis-associated ILD. Additionally, the small patient cohort limited the clinical application of these findings; therefore, a larger patient population should be examined using a prospective study model in future investigations.

## Conclusions

In conclusion, this study demonstrated increased HNP levels in the plasma and BALF of patients with myositis-associated ILD as compared with that observed in those of healthy controls. Although factors were associated with clinical parameters, further studies are necessary to clarify whether HNPs represent a candidate disease marker and to elucidate the role of defensins in myositis-associated ILD.

## Abbreviations

ARS: Aminoacyl-tRNA synthetase
BALF: Bronchoalveolar lavage fluid
CADM: Clinically amyopathic dermatomyositis
DM: Dermatomyositis
FDR: False-discovery rate
HNPs: Human neutrophil peptides
HRCT: High-resolution computed tomography
IIMs: Idiopathic inflammatory myopathies
ILD: Interstitial lung disease
IP: Immunoprecipitation
IQR: Inter-quartile range
KL-6: Krebs von den Lungen 6
LDG: Low-density granulocyte
LDH: Lactate dehydrogenase
MDA5: Melanoma-differentiation-associated protein 5
NET: Neutrophil extracellular trap
PM: Polymyositis
SP: Surfactant protein
